# Immunoaffinity purification and characterization of mitochondrial membrane-bound D-3-hydroxybutyrate dehydrogenase from *Jaculus orientalis*

**DOI:** 10.1186/1471-2091-9-26

**Published:** 2008-09-30

**Authors:** Driss Mountassif, Pierre Andreoletti, Zakaria El Kebbaj, Adnane Moutaouakkil, Mustapha Cherkaoui-Malki, Norbert Latruffe, M'hammed Saïd El Kebbaj

**Affiliations:** 1 INSERM U866 (Institut National de la Santé et de la Recherche Médicale), Université de Bourgogne, LBMC (Biochimie Métabolique et Nutritionnelle), Faculté des Sciences, 6 Bd Gabriel, 21000 Dijon cedex, France; 2 Laboratoire de Biochimie et Biologie Moléculaire, Université Hassan II – Aïn Chock, Faculté des Sciences Aïn Chock, km 8 route d'El Jadida BP. 5366, Mâarif, Casablanca, Morocco; 3 Laboratoire de Physiologie et Génétique Moléculaire, Université Hassan II – Aïn Chock, Faculté des Sciences Aïn Chock, km 8 route d'El Jadida BP. 5366, Mâarif, Casablanca, Morocco; 4 Unité de Radio-Immuno-Analyse, Département des Applications aux Sciences du Vivant, CNESTEN (Centre National de l'Energie, des Sciences et des Techniques Nucléaires), BP 1382 RP, 10001 Rabat, Morocco

## Abstract

**Background:**

The interconversion of two important energy metabolites, 3-hydroxybutyrate and acetoacetate (the major ketone bodies), is catalyzed by D-3-hydroxybutyrate dehydrogenase (BDH^1^: EC 1.1.1.30), a NAD^+^-dependent enzyme. The eukaryotic enzyme is bound to the mitochondrial inner membrane and harbors a unique lecithin-dependent activity. Here, we report an advanced purification method of the mammalian BDH applied to the liver enzyme from jerboa (*Jaculus orientalis*), a hibernating rodent adapted to extreme diet and environmental conditions.

**Results:**

Purifying BDH from jerboa liver overcomes its low specific activity in mitochondria for further biochemical characterization of the enzyme. This new procedure is based on the use of polyclonal antibodies raised against BDH from bacterial *Pseudomonas aeruginosa*. This study improves the procedure for purification of both soluble microbial and mammalian membrane-bound BDH. Even though the *Jaculus orientalis *genome has not yet been sequenced, for the first time a D-3-hydroxybutyrate dehydrogenase cDNA from jerboa was cloned and sequenced.

**Conclusion:**

This study applies immunoaffinity chromatography to purify BDH, the membrane-bound and lipid-dependent enzyme, as a 31 kDa single polypeptide chain. In addition, bacterial BDH isolation was achieved in a two-step purification procedure, improving the knowledge of an enzyme involved in the lipid metabolism of a unique hibernating mammal. Sequence alignment revealed conserved putative amino acids for possible NAD^+ ^interaction.

## Background

The NAD^+^-dependent D-3-hydroxybutyrate dehydrogenase (BDH: EC 1.1.1.30), which has been studied by our group for several years [1-9], plays a key role in redox balance and energy metabolism since it reversibly converts 3-hydroxybutyrate into acetoacetate (the two major ketone bodies largely produced under high lipolysis, diabetes, or fasting). In eukaryotic cells, BDH is a mitochondrial inner membrane-bound enzyme [1,10,11] and its active site is located on the matrix side [2,12]. BDH is coded by a nuclear gene and is synthesized in free cytosolic polysomes as a precursor that is posttranslationally imported into mitochondria and then processed at its N-terminus presequence [4,13]. A very unique property, the catalytic activity of the enzyme is lecithin-dependent [14,15]. The purified BDH is nonactive in absence of lipids but can insert spontaneously and unidirectionally into liposomal-phospholipid vesicles or into purified membranes and then become catalytically active [12]. It has previously been proposed that specific activation of BDH by phosphatidylcholine (PC)-containing liposomes involves an allosteric mechanism [16] in which PC enhances coenzyme-binding [17]. As reported by Williamson et al. [18], according to the equilibrium constant, in the presence of NADH, the hepatic BDH transforms acetoacetate into D-3-hydroxybutyrate, which is then transported through the blood stream to peripheral tissues, i.e., brain, heart, kidney, etc. In extrahepatic tissues, BDH catalyzes the reverse reaction where acetoacetate is used, after its conversion to acetyl-CoA, in ATP production. On the other hand, acetoacetyl-CoA can be used for fatty acid synthesis. A catalytic mechanism involving cystenyl and histidyl residues of the BDH active site for the interconversion of D-3-hydroxybutyrate and acetoacetate in both liver and peripheral tissues has been previously proposed by our group [7].

In striking contrast to mammalian BDH, the bacterial BDH is a cytosolic soluble enzyme and does not require phospholipids for its activity [19]. Indeed, the role of BDH in many bacteria is to produce D-3-hydroxybutyrate, which is a substrate for the synthesis of poly -3-hydroxybutyrate (PHB) as intracellular carbon energy storage [20].

Elsewhere, our group has long been interested in the lipid metabolism of an intriguing mammalian species: the jerboa (*Jaculus orientalis*) [9,21]. The jerboa is a nocturnal herbivorous rodent living mainly in Morocco's subdesert highland. It is an appropriate organism to study metabolic regulation because of its remarkable tolerance to heat, cold, dryness and scarce diet. This animal is a true hibernator [22], developing a seasonal obesity by accumulating fat during the prehibernation period. This fat is used during the hibernation period, together with carbohydrates, to produce energy via the formation of D-3-hydroxybutyrate by BDH [21].

To further characterize BDH from jerboa, it appeared necessary to overcome its low specific activity in mitochondria by purifying the enzyme from liver of the jerboa by establishing a new and original purification technique. Indeed, while bacterial BDH can be easily purified with the classical method for soluble enzymes [23,24], enormous effort has gone into purifying the mitochondrial membrane-bound BDH from mammals, mostly from bovine heart [1,6,25-34], rat liver [1,6,31-33], rat brain [34], recombinant rat liver enzyme expressed in *Escherichia coli *[35], and *Camelus *liver [8]. Typically, after membrane disruption by detergent (cholate or Triton X-100) or by phospholipase A2-generated lysophospholipids, the purification procedures were based on combined chromatographies (adsorption, dihydroxyapatite, ionic exchange, hydrophobic, NAD^+ ^or NAD^+^-related affinity, and often controlled pore glass beads). Unfortunately, these methods were difficult to adapt to other sources. Until now, no-one has proposed an immunoaffinity purification method. Here, we report the development of an antibody-antigen procedure based on the existence of conserved epitopes between bacterial and mammalian BDH. Indeed, BDH from *Jaculus orientalis *was purified using polyclonal antibodies raised against a prokaryotic BDH purified from the bacterium *Pseudomonas aeruginosa*. After solubilization of mitochondrial membranes using Triton X-100, purification of jerboa liver BDH was processed using ammonium sulfate precipitation and phenyl-Sepharose and Sepharose-Blue chromatographies. Final purification was achieved by immunochromatography, providing a 31 kDa single polypeptide chain. Moreover, even though the genome of *Jaculus orientalis *has not been sequenced, a D-3-hydroxybutyrate dehydrogenase cDNA from jerboa was cloned and sequenced for the first time. Sequence alignment revealed conserved putative essential amino acids for NAD^+ ^interaction. This study applied immunoaffinity chromatography to purify BDH, a membrane-bound and lipid-dependent enzyme. In addition, bacterial BDH was isolated in a two-step purification procedure, providing better knowledge of a lipid metabolism enzyme in a unique hibernating mammalian species.

## Results

### - Purification of soluble BDH from *Pseudomonas aeruginosa*

BDH was purified to electrophoretic homogeneity from *P. aeruginosa *extract in a two-step ammonium sulfate fractionation (27–42%) procedure, followed by Blue Sepharose CL-6B chromatography.

In a typical experiment, a total amount of 4600 mg of protein, corresponding to 1012 units of BDH, was obtained from crude extract of *P. aeruginosa*. After ammonium sulfate fractionation, the concentrated enzyme solution was applied to a Blue Sepharose CL-6B column. A specific activity of 11.2 U/mg of protein was obtained for the purified enzyme, with a yield of 6.6% and a purification factor of 50 (not shown).

The SDS-PAGE analysis of the different fractions obtained during this purification shows only one protein band at 29 kDa in the final enzyme preparation [Additional file 1].

Using purified BDH as the immunogen, we produced rabbit polyclonal antibodies, which selectively recognize a single immunoreactive band (29 kDa) in both crude extracts and purified preparations (not shown).

The polyclonal antibodies produced were purified and fixed to CN-Br Sepharose in order to purify the BDH from jerboa liver.

### - Purification of membrane-bound BDH from jerboa liver

In a typical trial, a total of 5100 mg of protein, corresponding to 5.5 units of BDH, was obtained after solubilization of mitoplast proteins using triton X-100 as nonionic surfactant. After ammonium sulfate fractionation, the concentrated enzyme solution was applied to phenyl-Sepharose HP, Blue Sepharose CL-6B, and immunoaffinity columns. Table 1 summarizes the results of the purification process. A specific activity of 0.030 U/mg of protein was obtained for the purified enzyme, with a yield of 0.50% and a purification factor of 37.

**Table 1:** Purification steps of BDH from jerboa liver

	**Total protein ** **(mg)**	**Specific activity ** **(nmol/min/mg of protein)**	**Total activity ** **(μmol/min)**	**Purification factor ** **(fold)**	**Yield ** **(%)**
**Crude extract**	5100	1.1	5.5	1.0	100
**Ammonium sulphate (30–50%)**	560*	8.2	4.5	7.4	82
**Phenyl-Sepharose**	350	10.4	3.6	9.4	65
**Sepharose-Blue**	50	17.6	0.87	16.0	16
**Immunoaffinity chromatography**	0.75	41.3	0.030	37.5	0.50

Typical experiments were reported from three independent trials.

* The ammonium sulphate precipitation step eliminates 91% of contaminating proteins from Jerboa crude extracts, respectively. These values were calculated from the total protein amount deduced from the amount of BDH (estimated from specific activities after the immunoaffinity chromatography step).

**Figure 1:**
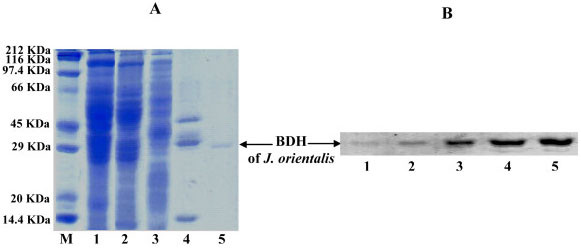
**BDH purification steps from jerboa liver**. Proteins (40 μg) were resolved by SDS-PAGE and stained with Coomassie Brilliant Blue (a) or subjected to Western blot (b) using the purified polyclonal anti-BDH antibodies. Lanes M, 1, 2, 3, 4, and 5 represent standard proteins, crude extract, 30–50% ammonium sulphate fraction, phenyl-Sepharose fraction, affinity chromatography fraction, and immunoaffinity chromatography eluate pool (pure protein preparation). Bound antibody was located by immunoreaction combined with peroxidase conjugated goat anti-rabbit IgG. The arrow (b) indicates the band corresponding to the BDH subunit.

The SDS-PAGE analysis shows that the immunoaffinity step is crucial to eliminate the remaining contaminants of the penultimate fractions. This last purification step shows a single 31 kDa protein (Figure 1), which has been described for other eukaryotic BDH subunits (Figure 1A, lane 5). The 31 kDa jerboa BDH monomer cross-reacts with the purified antibacterial BDH antibodies (Figure 1B).

### - Properties of the purified BDH from jerboa liver

BDH kinetic parameters of purified BDH from jerboa liver in liposome-reconstituted phospholipid-enzyme complex were determined. The results obtained show a value of 51 nmol/min/mg for V_max_, 0.45 mM, 2.1 mM, and 1.45 mM for K_M_NAD^+^, K_M_BOH and K_D_NAD^+^, respectively. The comparison of these values with the parameters of the native BDH bound to the inner mitochondrial membrane [9] shows small differences in the K_M _values. This can be explained by the fact that the purified BDH released from its mitochondrial membrane environment was successfully reconstituted in an active form following addition of mitochondrial phospholipids.

The effect of temperature on the BDH activity was followed. The results obtained show that the optimal temperature for the BDH activity is 35°C for *J. orientalis *[Additional file 2]. This is close to 37°C for BDH from *Camelus dromedaries *[8] but very different (55°C) for microbial BDH from *Acidovorax *[24].

Interestingly, like membrane-bound enzyme, the Arrhenius plots of the reconstituted active purified BDH show a break at 17°C [Additional file 3]. BDH activity dependence on temperature discontinuity was previously found for native BDH in the heavy mitochondria fraction from Jerboa liver [9]. This property is considered to reflect that BDH lipids depend on the physical state of the membrane phospholipid bilayer.

The optimal pH value of BDH activity is 8 [Additional file 4]. Similar results were found for rat [14], *Camelus dromedaries *[8], and for the bacteria *Acidovorax, Rhodospirillum rubrum *and *Rhodopseudomonas spheroides *[24].

### - Nucleotide sequence and analysis of *J. orientalis *BDH cDNA

**Figure 2:**
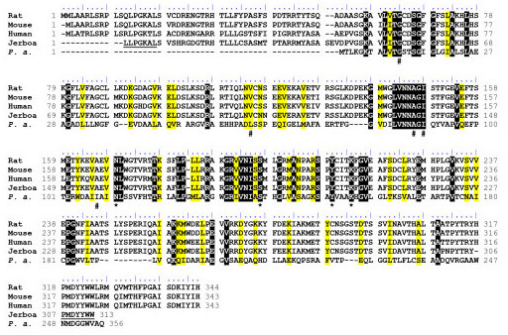
**Alignment of BDH sequences**. Alignment of BDH sequences from mammalian species (rat, mouse, human and jerboa) with *Pseudomonas aeruginosa *(*P. a.*) was realized using ClustalW (Thompson et al., 1994). Identical and similar residues were shown in black and yellow background respectively. The presumed amino acids sequences corresponding to oligonucleotides used for the PCR amplification of Jerboa BDH cDNA are underlined. According to the identity between Rat, Mouse and Human, they are considered as putative sequences in Jerboa. Amino acids of the catalytic tetrad Asn111, Ser139, Tyr152 and Lys156 (*P. a. *numbering) are marked by a star (*). These amino acids correspond to Asn114, Ser142, Tyr155 and Lys159 of the *Pseudomonas fragi *BDH (Ito et al., 2006). Amino acids participating to the NAD^+ ^binding pocket Gly12, Leu61, Ala88, Ile90 and Ile108 (*P. a. *numbering) are marked by a hash sign (#). These amino acids correspond to Gly11, Leu64, Ala91, Ile93 and Leu113 of the *Pseudomonas fragi *BDH (Ito et al., 2006).

In order to clone the cDNA encoding BDH from jerboa liver, RT-PCR, primers were selected from two highly conserved BDH regions (LPGKALS and PMDYYWW) from mammalian species since the jerboa genome has not yet been sequenced. For the nucleotide sequence, see the section titled "Method" section. The amplification procedure revealed a single cDNA fragment with the expected size (936 pb) [Additional file 5]. The sequenced clone (GenBank accession # bankit 1072824 EU563473) was aligned and compared with other BDHs, from several species, including the mammalian vertebrate phyla and bacterial species, using the BioEdit program [36]. The highest identity was shown when the sequence was aligned with other mammalian BDH sequences (human, rat and mouse). Indeed, the analysis shown in Figure 2 reveals 79% identity with rat and mouse, 75% with human and only 19% with *P. aeruginosa*. Jerboa BDH sequence is 92% complete since amino acids from the C-terminal side are not yet available. The differences in sequences obtained between mammalian and bacterial BDHs can be related to the biochemical properties of both enzymes since mammalian BDH is membrane-bound and located in mitochondria and bacterial BDH is soluble and cytosolic. Moreover, the comparison between the two BDH types in terms of cDNA-deduced sequences reveals the major difference in the length of the polypeptide chain: 343 amino acids for the human BDH vs 256 for *Pseudomonas*. The longer sequence of the mammalian enzyme is related to the mitochondrial targeting presequence at the N-terminus and to the phospholipid-binding region at the C-terminus (Figure 2) [3,37]. The sequence alignment shows 48 identical amino acids and 42 similar amino acids between the mammalian and the bacterial enzymes.

## Discussion and conclusion

Purifying BDH from Jerboa liver made it possible to overcome its low specific activity in mitochondria for further biochemical characterization of the enzyme.

Previous BDH purification procedures, partial or complete, were successively proposed by different groups in order to improve the purity, the stability, the yield, the time required or simplicity, and to adapt the technique to BDH from various mammalian sources. The purification procedures were often based on several chromatography steps by combining adsorption, hydrophobic, ionic exchange, or NAD^+ ^(or NAD^+^-related affinity such as the dye affinity matrix or controlled pore glass beads). The published procedures were not convenient to Jerboa liver BDH purification. For instance, rat liver [31] and bovine heart [27] BDH was not pure and/or contained significant amounts of residual phospholipids. The technique developed in Fleischer's lab [28,29], using controlled pore glass beads (CPG), was adapted for large-scale use and required a huge amount of starting biological material but provided a low yield (0.02%).

Our new procedure was based on the use of polyclonal antibodies raised against BDH from bacterial *Pseudomonas aeruginosa*. After purification steps using phenyl Sepharose and Blue-Sepharose, Jerboa liver BDH fractions were not pure to homogeneity and required an immunoaffinity column to achieve purification, yielding 0.5%.

The molecular weight of the purified jerboa BDH subunit (31 kDa) shows a similar value to the values given for most of the eukaryotic BDH, e.g., for bovine heart [30], rat liver [33] and human heart [38]. In contrast, BDH from *Camelus dromedaries *shows a molecular weight of 67 kDa [8], possibly corresponding to an evolutionary duplicated form. The primary sequence of BDH was previously determined for rat liver [39] and human heart enzyme [38].

The purified jerboa liver BDH from the BDH-antibody complex is in a readily reactivating form, since the active BDH-mitochondrial phospholipid complex shows similar enzymatic parameters as the native mitochondrially bound BDH, i.e., similar kinetic parameters, a break in the Arrhenius plot, optimum pH, and optimum temperature.

While the sequenced genome of *Jaculus orientalis *is not available, for the first time a BDH cDNA from jerboa has been cloned and sequenced. From: this and from the purified protein we assume that both correspond to the same molecular entity despite the fact that two kinetically different BDH enzymes were revealed in heavy and light mitochondria fromm jerboa liver [42]. Sequence alignment revealed putative essential amino acids for the NAD^+ ^interaction. The full identification and the spatial position of BDH strategic amino acids could not be achieved with a mammalian BDH since no 3D-structure is thus far available despite a number of attempts to obtain crystals (most particularly in studies on the bovine mitochondrial membrane-bound enzyme from S. Fleischer's group, Vanderbilt University, Nashville TN, personal communication). The available structural data are related to the structure of the bacterial BDH of *Pseudomonas fragi *(the only crystallized and modeled BDH [40]). Based on the *Pseudomonas fragi *BDH structure, modeling has revealed that conserved amino acids are closely localized to the BDH active site[40]. This analysis highlights the importance of these amino acids in the enzyme reaction, especially the strictly conserved tetrad: Asn114, Ser142, Tyr155 and Lys159 (amino acid numbers corresponding to *Pseudomonas fragi *BDH). In addition, Ito et al. [40] reported that the adenine of NAD^+ ^is accommodated in the hydrophobic pocket including Gly11, Leu64, Ala90, Ile93 and Leu113 (*Pseudomonas fragi *BDH). All these residues were also found in the BDH sequences studied (Figure 2).

This study applied immunoaffinity chromatography to purifying BDH, a membrane-bound and lipid-dependent enzyme. In addition, bacterial BDH isolation was achieved in a two-step purification procedure. This method also improved the knowledge of a lipid metabolism enzyme in a unique hibernating mammal.

## Methods

### - Microorganisms and growth conditions

Bacteria *Pseudomonas aeruginosa *(Pasteur Institute, Casablanca, Morocco) were grown aerobically at 37°C without exceeding the exponential phase in nutrient broth (Topley House, Bury, UK). The exponential phase was determined spectrophotometrically at 600 nm. The culture was inoculated with 1% (v/v) overnight preculture in the same medium.

### - Buffers

Buffer A: 50 mM potassium phosphate buffer (pH 7.5) containing 2 mM EDTA and 1 mM DTT.

Buffer B: buffer A containing ammonium sulfate at 50% saturation.

### - Crude extract preparation

Bacterial culture (5 l) was harvested by centrifugation at 2500 *g *for 10 min, washed three times with 50 mM potassium phosphate buffer (pH 7.5), and suspended in the same buffer containing 2 mM EDTA and 1 mM DTT (buffer A). Cells were disrupted at 4°C by sonication (30 s, 90% output, 12×) using a Bandelin Sonopuls sonifier. Cellular debris and unbroken cells were removed by centrifugation at 2500 *g *for 45 min at 4°C. The supernatant obtained constituted the crude bacterial extract (soluble protein fraction).

### - BDH purification from the bacterium *Pseudomonas aeruginosa*

The enzyme was purified from the crude bacterial extract in two steps: ammonium sulfate fractionation and Blue Sepharose CL-6B chromatography. All steps were performed at 4°C.

#### Ammonium sulfate fractionation

The crude extract of *P. aeruginosa *was subjected to protein precipitation in the 27–42% saturation range of ammonium sulfate at 4°C. The final pellet was dissolved in a minimal volume of buffer A. The protein solution was dialyzed overnight against 5 l of the same buffer.

#### Blue Sepharose CL-6B chromatography

The dialyzed enzyme preparation was applied to a Blue Sepharose CL-6B column equilibrated with two bed volumes of buffer A at 4°C. The column was washed with three bed volumes of buffer A. Finally, the enzyme was eluted with buffer A containing 0.1 mM NAD^+ ^at a flow rate of 6 ml/h. Active fractions were collected and conserved in 50% (v/v) glycerol at -20°C until use.

### - Production and purification of the anti-BDH antibodies against soluble BDH from *Pseudomonas aeruginosa*

A 1.5-kg New Zealand white rabbit, grown in the university's animal care facilities, was injected with 1 mg of the BDH purified from P. aeruginosa in aqueous solution (v/v) with incomplete Freund's adjuvant. After 21 days, a second dose of 800 μg of BDH was injected. After the 4th week, a third dose of 500 μg was again injected. One week later, the rabbit was anesthetized and 50 ml of blood were collected. The serum was separated after an overnight coagulation at 4°C and subsequent centrifugation.

#### Ammonium sulfate precipitation

The resulting serum, containing monospecific anti-BDH polyclonal antibodies, was brought to 40% saturation with solid ammonium sulfate ((NH_4_)_2_SO_4_), stirred for 1 h, and then centrifuged at 2500 *g *for 45 min. Afterwards, the pellet was dissolved in a minimal volume of phosphate buffer saline (PBS), pH 7.4, containing 137 mM NaCl, 2.7 mM KCl, 1.5 mM KH_2_PO_4_, and 4.3 mM K_2_HPO_4_. The antibody solution was dialyzed overnight against 5 l of the same buffer.

#### Ion-exchange chromatography

The dialyzed antibody preparation was applied at a flow rate of 6 ml/h to a DEAE-cellulose (Serva, Heidelberg, Germany) column (3 × 12 cm) that had been equilibrated with PBS. The column was extensively washed at the same flow rate with equilibrating buffer solution. Two-milliliter fractions were collected and those containing the anti-BDH antibodies were pooled. Since anti-BDH antibodies are iso-ionic at pH 7.4, they were not retained by the DEAE-cellulose and were generally left with the column's dead volume.

#### Immunoaffinity chromatography preparation

Sequence alignments from different species, including *P. aeruginosa*, human, rat, and mouse, revealed that BDHs share an amino acid identity between regions (LVNNAGI, VNI, PG). This property had prompted us to use the antibodies against bacterial BDH to purify the eukaryotic antibody.

We verified the specificity of anti-BDH antibodies by showing that BDH activities were completely inhibited in both *P. aeruginosa *and jerboa liver using immune serum, which did not inhibit BDH activity in jerboa GAPDH (glyceraldehyde-3-phosphate dehydrogenase) (data not shown). Moreover, preimmune serum had no effect. On the other hand, anti-BDH antibodies reacted with BDHs in western blotting (data not shown).

Immunoaffinity chromatography column (1 × 10 cm) was prepared with CN-Br Sepharose (Pharmacia) coupled with purified BDH from *P. aeruginosa *according the supplier procedure. After loading total polyclonal antibodies, the specific anti-BDH antibodies were eluted. and subsequently bound to CN-Br Sepharose in order to purify the BDH from jerboa liver with the same procedure as described above.

### Purification of mitochondrial membrane-bound BDH from jerboa liver

Jerboa housing: adult greater Egyptian jerboas (*Jaculus orientalis*, Rodentia, Dipodidae) (120–150 g, 4–6 months old) were captured in the area of Engil Aït Lahcen (in the subdesert eastern Morocco highland). They were adapted to laboratory conditions for 3 weeks at a temperature of 22°C with a diet of lettuce and rat chow and water ad libitum before killing. The light cycle during the experiment was set to 14 h of light and 10 h of darkness. Animal studies were conducted in accordance with the ethical recommendations on Animal Use and Care of the University Hassan II Casablanca.

Remark. We abandoned to purify liver BDH from hibernation Jerboa since hibernation is a complex and very difficult phenomenon to experimentally control and reproduce in a laboratory [9,21]. The rate of success is only 20% survival in contrast with active Jerboa housing.

#### Liver mitochondria and mitoplast isolation

The jerboas were decapitated and the livers (75 g total) were rapidly removed for mitochondria purification according to the technique described by Fleischer et al. [41] and as previously used by Mountassif et al. [42]. This method can be used to prepare high-yield mitochondria. The mitoplasts (outer membrane-free mitochondria) were prepared according to Kielley et al. [43]. Briefly, liver mitochondria were swelled in a 20-mM phosphate buffer at 0.5 ml/mg of protein for 30 min at 0°C. The mitoplasts were pelleted by centrifugation at 2500 g for 30 min.

#### Membrane solubilization and BDH release

The mitoplast fraction was dissolved in an equivalent volume of buffer A containing 0.2% Triton X-100 and then sonicated. The solubilization was complete after 1 h incubation on ice. The mixture was then centrifuged at 2500 *g *for 1 h and the supernatant containing the solubilized enzyme was collected.

#### Ammonium sulfate fractionation

The supernatant was subjected to protein precipitation in the 30–50% saturation range of ammonium sulfate. The final pellet was dissolved in a minimal volume of the buffer A containing ammonium sulfate at 50% saturation.

#### Phenyl Sepharose chromatography

The ammonium sulfate fraction was applied at the low flow rate (12 ml/h) to a phenyl Sepharose HP (Pharmacia Biotech) column (1.6 × 18 cm) pre-equilibrated with buffer B (buffer A containing ammonium sulfate at 50% saturation). After flow-thorough washing, the column was subjected to a decreasing linear gradient of ammonium sulfate (from 50% to 0%) in buffer A. The 5-ml fractions of the activity peak were pooled and dialyzed for 2 h against buffer A after addition of Triton X-100 to the 0.02% final concentration.

#### Blue Sepharose CL-6B chromatography

The dialyzed enzyme preparation was applied to a Blue Sepharose CL-6B column equilibrated with two bed volumes of buffer A. The column was washed with three bed volumes of buffer A. Finally, the enzyme was eluted with buffer A containing 10 mM NAD^+ ^at a flow rate of 6 ml/h. Active fractions were collected and pooled.

#### Immunoaffinity chromatography

For preparation (see the section titled "Production and purification of the anti-BDH antibodies against soluble BDH from *Pseudomonas aeruginosa*" above), BDH from jerboa liver was eluted by 5 M MgCl_2_, pH 7. Active fractions were selected by measuring the BDH activity level, collected and dialyzed at 4°C for 2 h against 5 l of buffer A containing 5 mM MgCl_2 _and 50% glycerol.

#### Phospholipid extraction and preparation of liposomes

Phospholipids were extracted from mitoplasts of jerboa liver according to Rouser and Fleischer [44]. One volume of mitoplast preparation was added to chloroform/methanol/0.8% KCl (13.3/6.7/4.2; v/v/v). The mixture was homogenized with an Ultraturrax at 7500 rpm for 3 min. After sedimentation, the organic phase was recovered and methanol/0.8% KCL/chloroform (48/47/3; v/v/v) was added. The chloroform phase was then concentrated in a rotary evaporator. The phospholipids were dissolved and sonicated in buffer A. The solution obtained was left to decant and the supernatant, which contains small liposomes, was stored at -20°C until use [45]. The amount of phospholipids was determined by measuring the phosphorus concentration according to Chen et al. [46]. Before use, the liposome preparation was quickly sonified.

#### Protein assay

The protein content was measured according to the Bradford procedure, using bovine serum albumin (BSA) as standard [47].

#### BDH reactivation

Purified BDH (10 μg) was incubated in the buffer containing 6 mM potassium phosphate, pH 8, 0.5 mM EDTA, 0.3 mM dithiothreitol in the presence of 0.2 μg mitochondrial phospholipid (estimated by lipid phosphorus determination). The mixture was incubated for 10 min at room temperature and enzymatic activity was then measured.

#### BDH activity determination

As described by El Kebbaj and Latruffe [7], BDH activity was measured at 37°C by monitoring NADH production at 340 nm (ε = 6.22 × 10^3 ^M^-1^cm^-1^) using 100 μg of protein homogenate (or 10 μg of purified enzyme) in a medium containing 6 mM potassium phosphate, pH 8, 0.5 mM EDTA, 0.3 mM dithiothreitol, in the presence of 2 mM NAD^+ ^(Sigma-Aldrich). The reaction was started by adding DL-3-hydroxybutyrate (Sigma-Aldrich) to the 10-mM final concentration.

### - Characterization of jerboa membrane-bound BDH

#### - Denaturing polyacrylamide gel electrophoresis

Sodium dodecyl sulfate polyacrylamide gel electrophoresis (SDS-PAGE) was performed as described by Laemmli [48] on one-dimensional 12% polyacrylamide slab gels containing 0.1% SDS.

### - Western blotting

After SDS-PAGE (12%) and subsequent transfer in nitrocellulose [49], the proteins (30 μg) were exposed to 1/100 dilution of monospecific polyclonal anti-BDH antibody and detected with the secondary antibody (anti-rabbit, IgG peroxidase conjugate) (Promega) diluted to 1/2500.

### - BDH enzymatic properties

Initial velocities were measured at varying BOH concentrations of (2.5–10 mM) or NAD^+ ^(0.5–2 mM). Michaelis constants (K_M_), dissociation constants (K_D_), and maximal velocity of the forward reaction were obtained by mathematical analysis following Cleland [50].

### - Determination of optimal pH and temperature-dependent BDH activity

The effect of pH on BDH activity was studied in a range from pH 4 to 10 using a mixture of different buffers (Tris, Mes, Hepes, potassium phosphate, and sodium acetate).

The temperature effects were characterized by activation and denaturation processes. For activation, the buffered medium containing 6 mM potassium phosphate, pH 8, and 0.5 mM EDTA was incubated for 2 min at temperatures from 5 to 80°C. Then, 2 mM of NAD^+ ^and 10 μg of purified BDH were added. The reaction was started immediately by the addition of 10 mM of BOH. For denaturation, 10 μg of purified BDH were incubated at temperatures from 5 to 80°C for 2 min in medium containing 6 mM potassium phosphate, pH 8, and 0.5 mM EDTA. Then 2 mM of NAD^+ ^were added and the enzymatic activity was measured by the later addition of 10 mM of BOH after 2 min of incubation at 37°C.

A BDH Arrhenius plot was obtained by measuring the enzymatic activity at temperatures from 5 to 40°C and interpreted as described by Raison [51].

### - RNA isolation and RT-PCR

Total RNAs were obtained from jerboa liver previously frozen in liquid nitrogen and stored at -80°C using Trizol reagent according to the supplier's protocol (Invitrogen).

The primers used were obtained from the alignment between consensus sequences of BDH from human, rat, and mouse.

First-strand cDNA was produced by reverse transcription (RT) using 200 units of Moloney Murine Leukemia Virus Transcriptase (Promega) in conjunction with 2 μg total RNA and the reverse primer; 5'-CCACCAGTAGTAGTCCATG-3' (corresponding to the LPGKALS amino acid sequence starting at amino-acid no. 13 in mouse and human BDH and at no. 14 in rat BDH) in a reaction mixture containing 50 mM Tris-HCl buffer, pH 8.3, 75 mM KCl, 3 mM MgCl_2_, 10 mM dithiothreitol, and 0.2 mM of each deoxynucleoside triphosphate for 1 h at 42°C. An aliquot from this template (1/10 of the reaction) was used in a subsequent polymerase chain reaction (PCR) using 1.25 U of GoTaq DNA polymerase (Promega), 0.04 μM of reverse and forward primer (5'-CTCCCAGGAAAA(A/G)C(C/T)CTAAGTG-3') (corresponding to the PMDYYWW amino acid sequence starting at amino acid no. 223 in mouse and human BDH and at no. 224 in rat BDH). PCR was performed for 35 cycles in the following conditions; 92°C for 30 s, 55°C for 30 s, and 72°C for 1 min 30 s.

### - Cloning and sequencing of the BDH clone from *J. orientalis*

The PCR product was purified using QIAEX II Kit (Qiagen) and subcloned into the pGEM-T vector system (Promega), and the nucleotide sequence was determined on both strands using universal primers T7 and SP6 (MWG Biotech, Germany).

The sequence obtained and other sequences were compared using the BioEdit program [36] and ClustalW [52].

## Abbreviations

BDH: D-3-hydroxybutyrate dehydrogenase; DL-BOH: DL-3-hydroxybutyrate; BSA: bovine serum albumin; EDTA: ethylene-diamine tetra-acetic acid; Hepes: 4-(2-hydroxyethyl)-1-piperazine ethane sulfonic acid; Mes: 4-N-morpholinoethanesulfonic acid; NAD(H): nicotinamide adenine dinucleotide oxidized/(reduced) forms; SDS-PAGE: sodium dodecyl sulfate-polyacrylamide gel electrophoresis; TMB: tetramethyl benzidine; Tris: trihydroxy-methyl-aminomethane.

## Authors 'contributions

DM had the original idea to purify BDH by immunoaffinity and conducted the purification and the enzyme characterization. PA managed the biochemical modeling and interpretation. ZEK contributed to Western blotting procedures. AM helped in the antibody preparation. MCM gave advise on the development of the paper and provided financial support. NL managed the work and improved the manuscript. MSEK, as general manager, assisted in bringing the project to term.

## Supplementary Material

Additional file 1 **Determination of BDH molecular weight in denaturing conditions of electrophoresis** Click here for file

Additional file 2 **Influence of temperature in the activation-denaturation processes of purified BDH from Jerboa** Click here for file

Additional file 3 **Arrhenius plot of Jerboa BDH activation catalytic process** Click here for file

Additional file 4 **pH dependency of Jerboa BDH activity** Click here for file

Additional file 5 **BDH cDNA clone amplification by RT-PCR** Click here for file
